# Saltelli Global Sensitivity Analysis and Simulation Modelling to Identify Intervention Strategies to Reduce the Prevalence of *Escherichia coli* O157 Contaminated Beef Carcasses

**DOI:** 10.1371/journal.pone.0146016

**Published:** 2015-12-29

**Authors:** Victoria J. Brookes, David Jordan, Stephen Davis, Michael P. Ward, Jane Heller

**Affiliations:** 1 Graham Centre for Agricultural Innovation, NSW Department of Primary Industries and Charles Sturt University, Pugsley Place, Wagga Wagga, NSW 2650, Australia; 2 School of Animal and Veterinary Sciences, Faculty of Science, Charles Sturt University, Wagga Wagga, NSW 2678, Australia; 3 NSW Department of Primary Industries, Wollongbar, NSW, Australia; 4 School of Mathematical and Geospatial Sciences, RMIT University, Melbourne, Victoria 3001, Australia; 5 Faculty of Veterinary Science, The University of Sydney, Camden, NSW 2570, Australia; Agricultural University of Athens, GREECE

## Abstract

**Introduction:**

Strains of Shiga-toxin producing *Escherichia coli O157* (STEC O157) are important foodborne pathogens in humans, and outbreaks of illness have been associated with consumption of undercooked beef. Here, we determine the most effective intervention strategies to reduce the prevalence of STEC O157 contaminated beef carcasses using a modelling approach.

**Method:**

A computational model simulated events and processes in the beef harvest chain. Information from empirical studies was used to parameterise the model. Variance-based global sensitivity analysis (GSA) using the Saltelli method identified variables with the greatest influence on the prevalence of STEC O157 contaminated carcasses. Following a baseline scenario (no interventions), a series of simulations systematically introduced and tested interventions based on influential variables identified by repeated Saltelli GSA, to determine the most effective intervention strategy.

**Results:**

Transfer of STEC O157 from hide or gastro-intestinal tract to carcass (improved abattoir hygiene) had the greatest influence on the prevalence of contaminated carcases. Due to interactions between inputs (identified by Saltelli GSA), combinations of interventions based on improved abattoir hygiene achieved a greater reduction in maximum prevalence than would be expected from an additive effect of single interventions. The most effective combination was improved abattoir hygiene with vaccination, which achieved a greater than ten-fold decrease in maximum prevalence compared to the baseline scenario.

**Conclusion:**

Study results suggest that effective interventions to reduce the prevalence of STEC O157 contaminated carcasses should initially be based on improved abattoir hygiene. However, the effect of improved abattoir hygiene on the distribution of STEC O157 concentration on carcasses is an important information gap—further empirical research is required to determine whether reduced prevalence of contaminated carcasses is likely to result in reduced incidence of STEC O157 associated illness in humans. This is the first use of variance-based GSA to assess the drivers of STEC O157 contamination of beef carcasses.

## Introduction

Strains of Shiga-toxin producing *Escherichia coli* O157 (STEC O157) are important foodborne pathogens in humans, causing a spectrum of syndromes ranging from inapparent infection and non-bloody diarrhoea, to haemorrhagic colitis and haemolytic uraemic syndrome (HUS) [1]. HUS has a case fatality rate of 5–10%, and other severe outcomes—including long-term renal impairment, seizures and hypertension—occur in some patients [2]. Although the incidence of STEC O157 associated illness is relatively low compared to the incidence associated with other foodborne pathogens [3], STEC O157 is the primary cause of HUS in several countries [4–7]. HUS is also more commonly associated with STEC O157 infection in children than adults, with approximately 15% of children with reported STEC O157 developing HUS [8]. As well as these potentially severe impacts on individuals, STEC O157 associated illness has broader economic impacts; Hoffman et al. [9] estimated the annual cost of STEC O157 associated illness in the United States (US) in 2009 at USD 254.8 million (90% credible range USD 25.1–1,102.5 million).

Cattle are asymptomatic hosts of STEC O157; the bacteria colonise the gastro-intestinal tract and are shed in faeces [10]. Outbreaks of disease in humans can arise from consumption of contaminated, undercooked beef or unpasteurised dairy products, and it is estimated that between 1982 and 2002 in the US, 21% and 2% of reported STEC O157 foodborne outbreaks were associated with beef and milk respectively [11]. Although STEC O157 colonisation has little, if any, impact on animal health, the economic cost and potential severity of illness in humans make STEC O157 a hazard of concern to cattle industries—particularly beef production—worldwide.

Prevention or reduction of STEC O157 contamination of beef is required to mitigate the risk of human illness, but despite extensive research, many aspects of STEC O157 ecology and epidemiology are still poorly understood and STEC O157 contaminated beef products remain an important cause of foodborne illness [2, 12–14]. A thorough understanding of the drivers of ecological systems such as that of STEC O157 can be difficult to achieve using empirical research due to both structural and dynamic complexity, as well as great variability and uncertainty associated with system inputs. In the case of STEC O157, this is further compounded by the relative low probability of human disease—necessitating large studies—and the existence of many strains of STEC O157 with different ecological and epidemiological characteristics [15].

Simulation of STEC O157 ecology and epidemiology using mathematical and computational modelling is an approach that can supplement empirical research. “Farm-to-fork” models have been used to study the entire chain to identify the drivers of disease in humans and although cooking temperature has consistently been found to be important, they have drawn different conclusions about the importance of other factors in the chain on human illness [16–18]. Most recently, a study by Smith et al. [19] found that combinations of interventions applied pre-harvest and throughout processing had the greatest impact on reducing the probability of illness. In addition to regional variation and uncertainty in input parameters, differences in the findings from “farm-to-fork” studies could be attributable to structural differences between models and methods for sensitivity analysis. Sections of the farm-to-fork chain have been examined using greater structural detail; for example, Kiermeier et al. [20] recently examined the drivers of human illness in the chain from processing to consumption of hamburgers made from Australian beef in the US. Cooking temperature again had the greatest influence on human illness, but the dispersion of STEC O157 in ground beef was also an important factor. The beef harvest chain was examined by Jordan et al. [21, 22] who determined that transfer of faeces from hide to carcass and hours of fasting prior to slaughter had the greatest influence on carcass contamination. This study was not conducted recently and given the findings of empirical research subsequently, re-assessment of the influence of factors in the beef harvest chain might identify more effective intervention strategies to reduce STEC O157 contamination of carcasses.

Sensitivity analysis is a key component of modelling studies to quantify the extent to which each input determines variability in model output [23]. Previous studies of the chain of events leading to STEC O157 associated illness in humans have used Spearman rank correlation coefficients—a type of global sensitivity analysis (GSA)–to identify important risk factors. This method assumes that linear relationships exist between each input and output of the system, and that interactions between inputs have negligible influence on the output. Results can be misleading in systems in which interactions between inputs are important. Variance-based methods for GSA allow for non-linear relationships between system inputs and the output and, in addition to providing an estimate of the individual importance of each input, they estimate of the importance of each input in combination with its interactions with other inputs. Variance-based GSAs have commonly been applied to ecological models, but rarely used in animal or human health studies [24]. Since STEC O157 ecology and epidemiology is a system in which non-linear relationships and interactions are likely to be important, variance-based GSA might improve understanding about the importance of drivers of STEC O157 contamination of beef carcasses.

The objective of the current study was to determine the most effective intervention strategies to reduce the potential maximum prevalence of STEC O157 contaminated beef carcasses produced in abattoirs. This was achieved by using a computational model of the beef-harvest chain and the Saltelli method, a variance-based GSA not previously applied to models of STEC O157 ecology and epidemiology [25–28]. The results of this analysis can be used to focus interventions at points in the harvest chain at which the greatest reduction in the prevalence of carcass contamination with STEC O157 can be achieved, and identify the most important information gaps for targeted research.

## Methods

### Overview of model structure and simulation process

**Fig 1:**
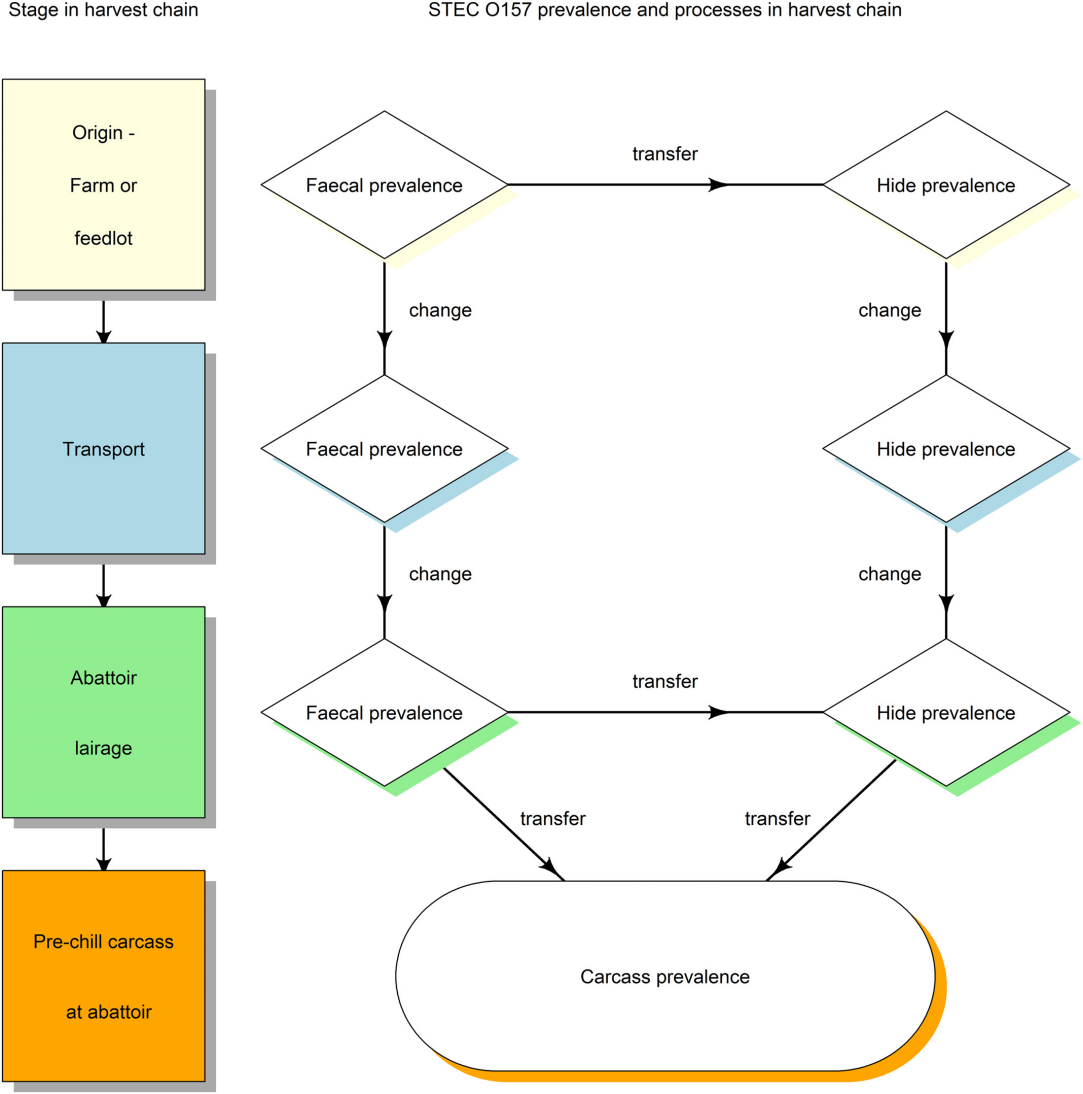
Structure of model to determine the most effective interventions to reduce *Escherichia coli* O157 (STEC O157) contamination of beef carcasses.

A stochastic, individual-animal-based, discrete-time computational model was constructed and implemented in the Python programming language (www.python.org, accessed 28/01/15). The model simulated the presence of STEC O157 in faeces, on hides and on carcasses of individual animals on a daily basis at the following stages in the harvest chain: origin (farm or feedlot), transport, lairage at the abattoir and slaughter up to stage of pre-chill carcass (Fig 1). Processes in the model that affected the status (positive or negative for STEC O157) of individual animal’s hide, faeces or carcass and resulted in changes to prevalence of STEC O157 on hides, in faeces or on carcasses were modelled stochastically using a mathematical approach described by Vynnycky and White [29], in which a random number was generated and the process only occurred if the random number was within the range specified for the input variable (alternatively known as a Bernoulli process). Processes included changes in individual animal’s faecal and hide status of STEC O157 between points from origin to lairage, and transfer of STEC O157 both from faeces to hides at origin and in the lairage, and from hides and faeces to pre-chill carcasses during the slaughter process. Processes and their parameters are listed in Table 1.

**Table 1:** Inputs and initial parameter ranges (maximum-range scenario) used in a simulation model to determine the most effective interventions to reduce *Escherichia coli* O157 (STEC O157) contamination of beef carcasses.

Parameter	Code	Range	Data source
**Origin (farm or feedlot)**			
Prevalence of cattle with STEC O157 in faeces	Prevalence_Faeces_Origin	1–100%	[13, 33]
Hide contamination with STEC O157 from faeces of infected cattle; exponential function, *r* parameter (see Eq 2)	Hide_Contamination_Origin	0–10.475	[34]
Duration of colonisation	Duration_colonisation	1–45 days	[35–37]
**Transport**			
Truck size	Truck_Size	2–65 cattle	authors’ assumption
Number of trucks from each farm or feedlot	Number_Trucks_From_Origin	1–30	authors’ assumption
Duration of travel	Days_Travelled	1–2 days	[32]
*R*_0_ (travel)	*R*_0__Travel	0–10.0	[35]
Change in hide contamination with STEC O157 (environmental factors)	Hide_Contamination_Travel	-0.2–0.5	authors’ assumption
Probability of new GIT colonisation with STEC O157 (environmental sources)	Truck_GIT_re-colonisation	0–0.1	authors’ assumption
**Lairage**			
Duration lairage	Duration_Lairage	1–5 days	authors’ assumption
*R*_0_ (lairage)	*R*_0__Lairage	0–20.0	[35]
Change in hide contamination with STEC O157 (environmental factors)	Hide_Contamination_Lairage	-0.7–0.7	authors’ assumption
Probability of new GIT colonisation with STEC O157 (environmental sources)	Lairage_GIT_ re-colonisation	0–0.2	authors’ assumption
Hide contamination with STEC O157 from faeces of infected cattle; exponential function, *r* parameter (see Eq 2)	Hide_Contamination_Lairage_GIT	0–10.475	[34]
**Abattoir**			
Number of farms and feedlots represented in each slaughter run	Number_Farms_Feedlots	1–30	authors’ assumption
Number of animals in each slaughter run (daily throughput)	Abattoir_Throughput	50–2500	[38]
Transfer from hide to carcass; linear transfer ratio	Hide_To_Carcass_Transmission	0.05–0.45	[4, 39]
Transfer from GIT to carcass; linear transfer ratio	GIT_To_Carcass_Transmission	0.35–0.9	[4, 39]

The main output of the model was the maximum prevalence of STEC O157 contaminated carcasses from the total cattle slaughtered on a single day at an abattoir. Prevalence of STEC O157 in faeces and on hides at each point in the harvest chain were also recorded and validity of the structure, dynamics and outputs of the model was assessed qualitatively during model construction according to the known epidemiology of STEC O157, industry practices during the harvest chain and expected outputs in the context of peer-reviewed literature. Mixing of cattle from multiple sources during travel and lairage, and movements involving saleyards and travel rest periods were not included to avoid structural complexity.

Initially, the model simulated a scenario in which input variables were selected from maximum parameter ranges (maximum-range scenario, Table 1) to obtain a range of potential daily prevalence of STEC O157 contaminated carcasses from abattoirs. This represented a “worst-case” scenario to which subsequent simulations with interventions could be compared. Model inputs that had the greatest influence on variance were identified by GSA using the Saltelli method. This guided the selection of interventions which were introduced by reducing parameter ranges of inputs with the greatest influence on output to reflect ranges expected with current or potential interventions (Table 2). Subsequent simulations tested the impact of interventions on reduction of the maximum prevalence of STEC O157 contaminated carcasses. GSA was repeated after each simulation to guide introduction of further interventions.

**Table 2:** Inputs and parameter ranges used in simulations with interventions in a model to determine the most effective interventions to reduce *Escherichia coli* O157 (STEC O157) contamination of beef carcasses.

	Simulated scenario (see table footnote)										
Parameter	1	2	3	4	5	6	7	8	9	10	11
**Origin (farm or feedlot)**											
Prevalence of cattle with STEC O157 in faeces (%)	**0–30**	**0–30**	0–100	0–100	0–100	0–100	0–100	**0–30**	**0–30**	**0–30**	**0–30**
Hide contamination with STEC O157; exponential function, *r* parameter	0–10.475	0–10.475	0–10.475	0–10.475	0–10.475	0–10.475	0–10.475	0–10.475	0–10.475	0–10.475	0–10.475
Duration of colonisation (days)	1–45	**1–15**	1–45	1–45	1–45	1–45	1–45	1–45	**1–15**	1–45	**1–15**
**Transport**											
Truck size	2–65	2–65	2–65	2–65	2–65	2–65	2–65	2–65	2–65	2–65	2–65
Number of trucks from each farm or feedlot	1–30	1–30	1–30	1–30	1–30	1–30	1–30	1–30	1–30	1–30	1–30
Duration of travel (days)	1–2	1–2	1–2	1–2	1–2	1–2	1–2	1–2	1–2	1–2	1–2
*R*_*0*_ (travel)	0–10	**1–1.5**	0–10	0–10	0–10	0–10	0–10	0–10	**1–1.5**	1–10	**1–1.5**
Change in hide contamination with STEC O157	-0.2–0.5	-0.2–0.5	-0.2–0.5	-0.2–0.5	-0.2–0.5	-0.2–0.5	-0.2–0.5	-0.2–0.5	-0.2–0.5	-0.2–0.5	-0.2–0.5
Probability of new GIT colonisation with STEC O157	0–0.1	**0–0.05**	0–0.1	0–0.1	0–0.1	0–0.1	0–0.1	0–0.1	**0–0.05**	0–0.1	**0–0.05**
**Lairage**											
Duration lairage (days)	1–5	1–5	1–5	1–5	1–5	1–5	1–5	1–5	1–5	1–5	1–5
*R*_*0*_ (lairage)	0–20	**0–3**	0–20	0–20	0–20	0–20	0–20	0–20	**0–3**	0–20	**0–3**
Change in hide contamination with STEC O157	-0.7–0.7	-0.7–0.7	-0.7–0.7	-0.7–0.7	-0.7–0.7	-0.7–0.7	-0.7–0.7	-0.7–0.7	-0.7–0.7	-0.7–0.7	-0.7–0.7
Probability of new GIT colonisation with STEC O157	0–0.2	**0–0.1**	0–0.2	0–0.2	0–0.2	0–0.2	0–0.2	0–0.2	**0–0.1**	0–0.2	**0–0.1**
Hide contamination with STEC O157; exponential function, *r* parameter	0–10.475	0–10.475	0–10.475	0–10.475	0–10.475	0–10.475	0–10.475	0–10.475	0–10.475	0–10.475	0–10.475
**Abattoir**											
Number of farms and feedlots represented in each slaughter run	1–30	1–30	1–30	1–30	1–30	1–30	1–30	1–30	1–30	1–30	1–30
Number of animals in each slaughter run (daily throughput)	50–2500	50–2500	50–2500	50–2500	50–2500	50–2500	50–2500	50–2500	50–2500	50–2500	50–2500
Transfer from hide to carcass; linear transfer ratio	0.05–0.45	0.05–0.45	**0–0.03**	0.05–0.45	**0–0.03**	**0–0.02**	**0–0.01**	**0–0.03**	**0–0.03**	**0–0.01**	**0–0.01**
Transfer from GIT to carcass; linear transfer ratio	0.35–0.9	0.35–0.9	0.35–0.9	**0–0.03**	**0–0.03**	**0–0.03**	**0–0.03**	**0–0.03**	**0–0.03**	**0–0.03**	**0–0.03**

Simulation scenario:

1. reduced prevalence of STEC O157 in faeces at origin

2. vaccination

3. reduced probability of hide to carcass transfer (0–0.03)

4. reduced probability of GIT to carcass transfer (0–0.03)

5. interventions 3 and 4: reduced probability of hide and GIT to carcass transfer (both 0–0.03)

6. reduced probability of hide (0–0.02) and GIT (0–0.03) to carcass transfer

7. reduced probability of hide (0–0.01) and GIT (0–0.03) to carcass transfer

8. interventions 5 and 1

9. interventions 5 and 2

10. interventions 7 and 1

11. interventions 7 and 2

The following sections describe model simulation and global sensitivity analysis using the Saltelli method, and model inputs and parameter ranges in greater detail.

### Model simulation and global sensitivity analysis

Variance-based GSA using the Saltelli method determined which inputs most influenced the variance of prevalence of STEC O157 contaminated carcasses in each simulation [23, 28]. The Saltelli method is an extension of the concept and method developed by Sobol’ [26, 27] and was implemented in this study using the SALib module in Python (Jon Herman et al., http://jdherman.github.io/SALib/, accessed 07/02/2015). The sequence of events for each simulation were: input parameter sampling to create a matrix of parameter sets for each iteration within a simulation, simulation using the parameter sets to obtain model output (prevalence of STEC O157 contaminated carcasses) and estimation of sensitivity indices (SIs) to apportion output variance to each input.

#### Parameter sampling and simulation

Input parameter ranges were sampled using the Sobol’ sequence for each simulation. This is a quasi-random sampling method designed to optimise simulation and analysis, and has been found more computationally efficient for analysis of SIs than other methods such as Latin hypercube sampling [30]. Sample size was 5000, resulting in a parameter set size—and subsequent number of model iterations—of 190,000 (according to Eq 1, where *I* = number of iterations in each simulation, *N* = sample size and *k* = number of model inputs). Sample size was limited to 190,000 due to computational power and the time taken for simulations of this size (5 days).

I=N(2k+2) (1)

#### Estimation of sensitivity indices (SIs)

Total and first-order SIs for inputs were estimated following each model simulation. First-order effect indices represent output variance attributable to each input without considering interactions with other inputs. Total effect indices represent the total contribution to output variance by each input, combined with all interactions. In this study, SIs were normalised by total output variance, and plotted as centipede plots with 95% confidence intervals. Model output is most sensitive to inputs with the highest indices. In non-additive models in which there are no interactions between inputs, the value of the sum of first-order and total effect SIs both equal one. Interactions between inputs are inferred if the total effect indices sum to greater than one (non-additive models). The relative influence of interactions in simulations can be measured by comparison of the sum of total effect indices.

Variance is a summary measure of the deviation of each observation from the mean, and is an indicator of the spread of observations. It should be noted that it is possible for the variance of an output variable to decrease even though the range is constant; this occurs when kurtosis (the proportion of observations within a certain distance of the mean) increases. Interventions that targeted inputs with high SIs might have little impact on the maximum output of the model for this reason. Also, the relationship between the input and the output might not be linear and reduction in maximum output might only be substantial with a parameter range lower than that of the intervention. Therefore, whilst Saltelli GSA identified inputs with the greatest influence on output variance and directed interventions, the efficacy of interventions on reducing the maximum potential prevalence of STEC O157 contaminated carcasses was tested in subsequent simulations.

### Model inputs and parameter ranges

Where possible, information from peer-reviewed literature and industry reports was used to parameterise the model (Table 1). In the case of conflicting or scant information, the authors made assumptions about ranges to represent a worst-case scenario. Parameter ranges were uniformly distributed. Saltelli sensitivity analysis assesses the variance of the output to the full range of the input variables; therefore, the use of uniform distributions does not limit this form of sensitivity analysis, and is advantageous in the case of limited information about the shape of distributions for inputs. The aim of this study was to determine the effect of input parameters on possible prevalence of contaminated carcasses, not to measure risk associated with input variables (the probability of prevalence of contaminated carcasses). Therefore, the outcomes of interest were the sensitivity indices for each input and the maximum possible prevalence of STEC O157 contaminated carcasses (the minimum possible prevalence was zero for all simulations) for each simulation rather than the most likely prevalence, and the use of uniform distributions did not limit the conclusions drawn from this study.

The duration in days of each iteration varied according to duration of travel and lairage (holding cattle in pens at the abattoir). Although the distance that Australian cattle travel to abattoirs can reach thousands of kilometres [31], the duration of transport for cattle over 6 months old must not exceed 48 hours, at which time a rest period of at least 36 hours is required before transport can be continued [32]. Therefore, the range of duration of travel was 1–2 days in the current model, with the first day simulating travel duration up to 24 hours to the abattoir (Days_Travelled, Table 1). Most cattle are slaughtered within the first day of arrival at an abattoir, but occasionally this is extended and cattle are penned with access to food and water. To reflect this, the range of duration of lairage at the abattoir used in this model was 1–5 days, with the first day simulating slaughter within 24 hours of arrival at the abattoir (Duration_Lairage, Table 1).

The number of cattle modelled in each iteration was determined by abattoir daily throughput and ranged from 50–2500 cattle (Abattoir_Throughput, Table 1). Within this range, a number of farms or feedlots was selected (1–10), with varying truck sizes (2–65 cattle) and number of trucks (1–30) from each farm or feedlot (Number_Farms_Feedlots, Truck_Size, Number_Trucks_From_Origin, Table 1).

#### Prevalence of cattle with STEC O157 in faeces

Recent longitudinal studies on two farms in Australia in which cattle were repeatedly sampled over 6–9 month periods identified great variation in apparent daily prevalence of cattle with STEC O157 in faeces (prevalence of faecal STEC O157), ranging from 9.6% to 94.3% in a herd of dairy heifers, and 0% to 56.5% in a herd of mature beef cattle [13, 33]. To reflect this wide range, 0–100% was used to parameterise the prevalence of STEC O157 in faeces for each farm or feedlot at origin, and individual cattle were stochastically assigned as positive or negative for faecal STEC O157 according to the farm or feedlot prevalence.

Results from studies to determine the effect of transport or abattoir lairage on the prevalence of faecal STEC O157 are inconclusive; some indicated increased prevalence, whilst others found no effect on prevalence [40–42]. Specific processes for colonisation or re-colonisation of cattle during transport and lairage have also not been identified. Therefore, wide parameter ranges were used in this study to allow the faecal STEC O157 status of individuals to change through colonisation or re-colonisation of cattle according to the following broad processes.

Transmission of STEC O157 from the environment to cattle during travel and lairage was assumed possible; STEC O157 has been isolated from the harvest-chain environment [41]. There is no information on the probability of colonisation of cattle from these sources. Therefore, the authors made the assumption that the probability of colonisation of individual cattle from the environment was 0–0.1 and 0–0.2 during transport and lairage respectively (Truck_GIT_re-colonisation and Lairage_GIT_re-colonisation, Table 1). The maximum probability was higher at the abattoir due to greater opportunity for individuals to move around and contact a larger surface area than during transport, and the results of a previous study that found that environmental samples from the abattoir had higher prevalence of STEC O157 than samples from trucks [41].

Direct transmission of STEC O157 between cattle was also assumed to occur and, consistent with previous studies, the infectious process in individual cattle was described using a "susceptible-infectious-susceptible" model [43, 44]. The duration of colonisation (Duration_colonisation, Table 1) ranged from 1–45 days to reflect both intermittent and long duration of shedding reported in previous studies [35–37]. The probability of transmission was calculated using the Reed-Frost formula according to the number of infectious individuals (positive faecal STEC O157 status) and the probability of effective contact between two individuals on each day, which was calculated according to *R*_0_, the number of animals in the truck or lairage, and the duration of colonisation [45]. *R*_0_ ranged from 0–10.0 and 0–20.0 during transport and lairage respectively (R0_Travel and R0_Lairage, Table 1). Maximum *R*_0_ (20) was estimated using maximum prevalence from Williams et al. [13], making assumptions that STEC O157 is transmitted directly between cattle and causes epidemic waves of colonisation [33]. Maximum *R*_0_ was lower during transport due to reduced opportunity for transmission to occur on trucks in which cattle movements are restricted. Although, the cattle in the study by Williams et al. [13] were dairy heifers aged 2 to 13 months old—therefore, not representative of the full age-range of cattle presented at abattoirs—the maximum *R*_0_ can be considered to reflect the potential for transmission between young animals (for example, veal calves) in the harvest chain.

#### Prevalence of cattle with STEC O157 contaminated hides

**Fig 2:**
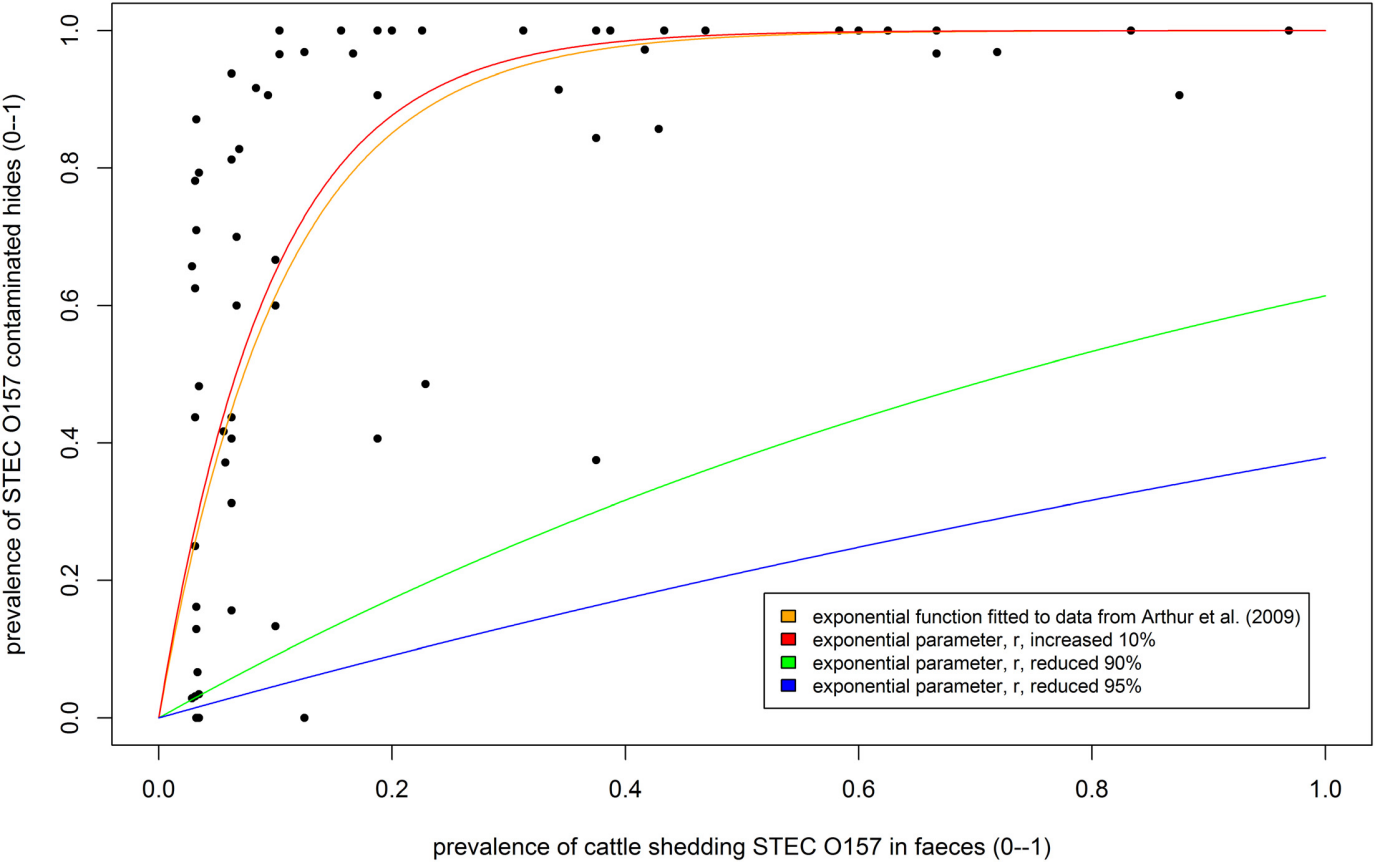
Relationship between prevalence of *Escherichia coli* O157 (STEC O157) in faeces and on hide. Orange line = exponential model (Eq 2) fitted to experimental data [34], fitted exponential parameter, *r* = 9.523; red line = 10% increase in fitted exponential parameter, *r*; green and blue line = 90% and 95% reductions in fitted exponential parameter, *r*.

A longitudinal study of cattle in feedlots in North America found that greater than 80% of hides were contaminated with STEC O157 when at least 20% cattle were shedding STEC O157 in faeces [34]. Data from this study were used to fit and define an exponential relationship between the prevalence of faecal shedding and contaminated hides (Fig 2), described by Eq 2 in which *H* denotes hide prevalence, *F* is the prevalence of cattle with STEC O157 in faeces, and *r* is exponential parameter selected for the iteration. Cattle in feedlots are relatively confined, and it has been suggested that cattle presented for slaughter from extensive farming systems have cleaner hides—and hence, an assumed lower prevalence of hide contamination with STEC O157 –than cattle from feedlots [46]. To account for a possible wide range of transfer ratios (including feedlots in which contamination might be worse), the exponential parameter used to describe the transfer of faecal STEC O157 to hides at the point of origin and during abattoir lairage in the current study ranged from 0–110% of the parameter derived from Arthur et al. [34] (Hide_Contamination_Origin, Hide_Contamination_Lairage_GIT; Table 1). The most probable mechanism for hide contamination is considered to be lying in contaminated faeces; therefore, hide contamination direct from faeces was not modelled during transport. Individual cattle were stochastically assigned positive or negative hide status according to the daily prevalence of STEC O157 in faeces at origin or in the lairage and the probability transfer of STEC O157 from faeces to hide (calculated according to Eq 2).

$$H=1-exp^{\left(-r\;F\right)}$$ (2)

It is difficult to determine the effect of transport and lairage on the prevalence of STEC O157 contaminated hides from empirical studies. For example, an Australian study found reduced prevalence of contaminated hides post-transport and in the lairage prior to slaughter [41], whilst a North American study found that cattle had an increased risk of hide contamination with increased time travelled and also when held in pens either contaminated with faeces or from which STEC O157 was isolated [47]. The latter study is consistent with findings of Arthur et al, [42], who also noted that only 29% of isolates identified at the abattoir matched those identified prior to transport. Due to this uncertainty and the lack of information on specific mechanisms for contamination of hides, the authors assumed wide ranges for the probability that an individual's hide status changed during transport and lairage (Hide_Contamination_Travel, Hide_Contamination_Lairage; Table 1). The range of probability of change in status during lairage was assumed wider than during transport due to greater movement and opportunity to contact a larger surface area by individual cattle in lairage, and the practice of washing cattle to clean hides prior to slaughter in some abattoirs. Negative ranges (transport -0.7–0, lairage -0.2–0) were used to represent the probability of change to negative hide contamination status for an individual with positive hide contamination status (a random number, range -1–0, was selected and return to negative hide status occurred if the random number was within the negative range specified for the input variable [29]). Conversely, positive ranges (transport 0–0.7, lairage 0–0.5) were used to represent the probability of change to positive hide contamination status for an individual with negative hide contamination status.

#### Transfer of STEC O157 from faeces and hides to carcasses

**Fig 3:**
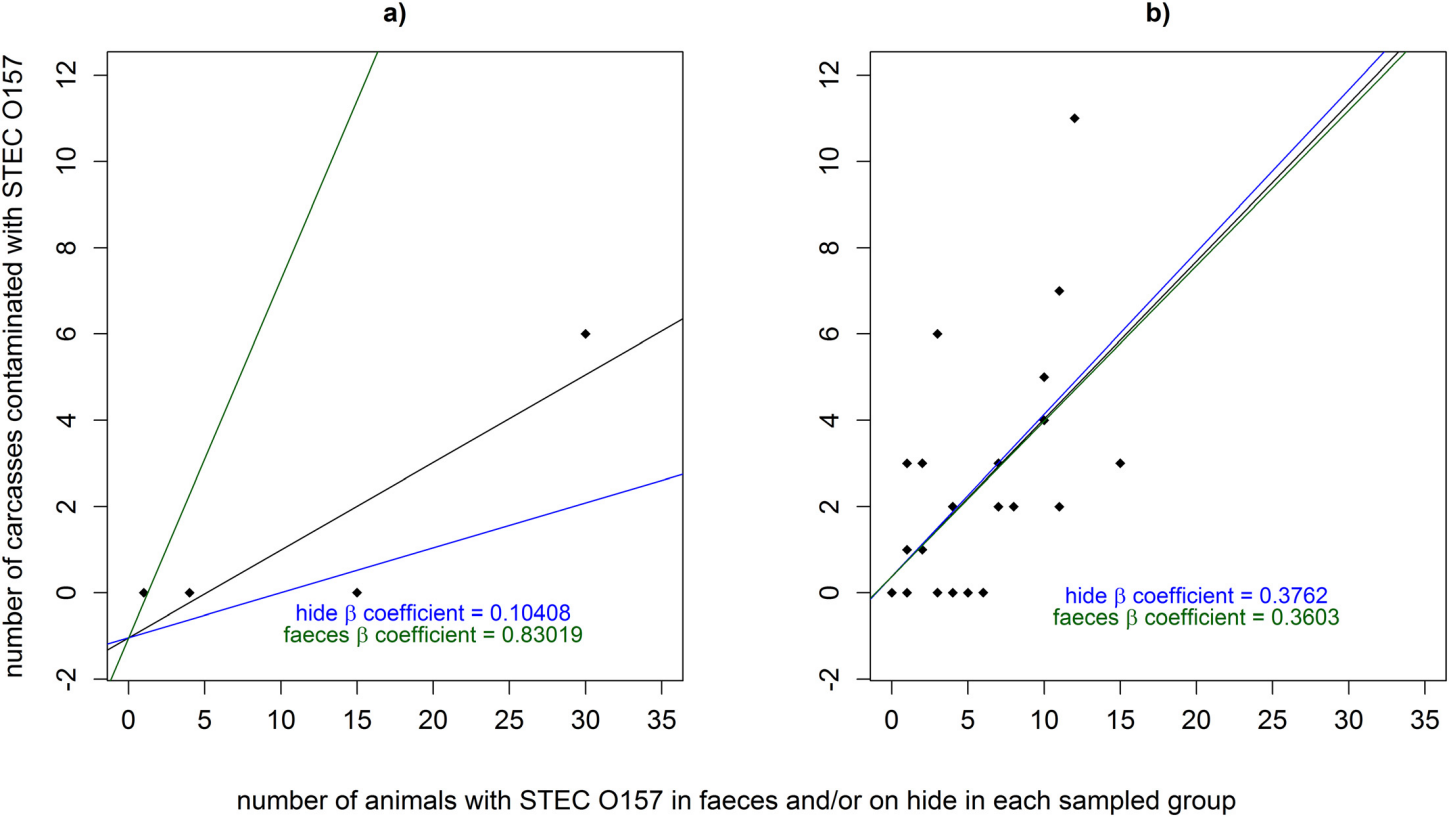
Relationship between number of animals with *Escherichia coli* O157 (STEC O157) both in faeces and on hide, and number of animals with STEC O157 contaminated carcasses; a) linear regression model fitted to data from a study in an abattoir in Australia (Fegan et al., 2005), b) linear regression model fitted to data from a study in four abattoirs in the United States (Elder et al., 2000). In each plot; black diamonds = data points from each study—a diamond shows the number of animals with positive faeces and/or hide (x axis) plotted against the number of animals in the group with positive carcasses (y-axis), black line = fitted linear regression line, blue line = hide β coefficient, green line = faeces β coefficient.

During the slaughter process, carcass contamination with STEC O157 can arise from transfer of STEC O157 from hides or faeces from the gastro-intestinal tract (GIT) [48]. Two studies were identified in which both faeces and hides were sampled during the slaughter process and from carcasses post-evisceration, prior to carcass decontamination processes and chilling [4, 49]. Data from these studies were used to parameterise the transfer of STEC O157 to carcasses in the current study. Although data from the study by Fegan et al. [49] were few and from one abattoir, results suggested that one animal with a high concentration of STEC O157 in faeces cross-contaminated a group of carcasses. Therefore, data from this study were used to account for the possibility that high concentrations of STEC O157 in faeces can overwhelm abattoir hygiene practices during slaughter, resulting in a higher prevalence of carcass contamination. Linear regression models were fitted to data from each of these studies to define the relationship between the number of animals identified with contaminated hide and faeces and the number of contaminated carcasses from each sampled group (Fig 3). Carcass contamination was described in the current model using minimum and maximum β coefficients (+/- standard error) from each study as linear transfer ratios from hide and faeces to carcasses (Hide_To_Carcass_Transmission, GIT_To_Carcass_Transmission; Table 1). Given the scant data to parameterise these inputs, the transfer ratios were not adjusted to account for the negative alpha coefficients associated with the linear regression models, thus making more conservative estimates of carcass contamination in this model.

### Intervention strategies

Following simulation of the initial scenario with all input parameters set in maximum range, further simulations tested the impact of interventions on reducing the maximum possible prevalence of STEC O157 contaminated carcasses. Introduction of interventions was guided by the results of repeated Saltelli GSA from previous simulations. Parameters with the highest total effect SIs—therefore, inputs with the greatest influence on output variance—were reduced to ranges that could be expected with current or potential industry intervention strategies. These strategies are described below and parameters for each intervention are presented in Table 2.

#### Reduction of prevalence of STEC O157 in faeces

A number of intervention strategies have been investigated and their impacts on prevalence of STEC O157 are reviewed by Thomas et al. [2] and Sargeant et al. [50]. Methods include vaccination (discussed below), manipulation of diet, probiotics, vitamin D, sodium chlorate, bacteriophages, antimicrobials, growth promoters, and improved farm hygiene practices. There is limited information on the use of any of these treatments in commercial settings. Some methods—sodium chlorate, probiotics and improved farm hygiene—reduced the prevalence of STEC O157 in faeces more consistently than other methods. Probiotics in feed had the largest reported effect on reduction of prevalence STEC O157 in faeces (69%, [51]). This is consistent with the findings of a recent systematic review [52], which determined that growing cattle who received direct-fed microbials had significantly lower prevalence of STEC O157 in faeces. In the current study an initial range of 0–30% was used for prevalence of cattle with STEC in faeces to represent the effect of introduction of this intervention.

#### Vaccination

A systematic review and meta-analysis found that some types of vaccine (Type III protein vaccines and siderophore receptor and porin protein vaccines) reduced prevalence of faecal STEC O157 in ruminants in both deliberate challenge and natural exposure trials [53]. In addition, Potter et al. [54] found that Type III protein vaccines reduced the duration of colonisation and reduced numbers of STEC O157 shed in faeces. To simulate vaccination in the current study, the range for prevalence of cattle with STEC O157 in faeces was reduced to 0–30% and the duration of colonisation was reduced to 1–15 days. The authors also made the assumptions that vaccination would reduce the probability of transmission between cattle during each time step—due to both fewer infected animals and lesser amounts of STEC O157 shed in faeces—and decrease the probability of colonisation from environmental sources. Therefore, vaccination simulation also incorporated ranges for *R*_0_ of 0–1.5 and 0–3, and probability of GIT re-colonisation (from environmental sources) were 0–0.05 and 0–0.1 during travel and in lairage respectively (Table 2).

#### Abattoir practices

Kiermeier et al. [46] stated that mean prevalence of *Escherichia coli* (not specifically STEC O157) contaminated beef carcasses at a sample abattoirs in Australia was 3.0% for steers or heifers, and 7.1% for cows or bulls. Samples in this study were taken post-processing when reduced prevalence of bacterial contamination is expected following processing interventions such as hot-water washing and steam pasteurisation of carcasses [55]. STEC O157 is a proportion of these isolates; therefore, in the current study, ranges of 0–0.03 were used for linear transfer ratios for STEC O157 carcass contamination from both GIT and hide to simulate implementation of interventions between slaughter and de-hiding and evisceration. Due to uncertainty about these parameters, further ranges of transfer ratios for these parameters were also tested (Table 2).

## Results

**Fig 4:**
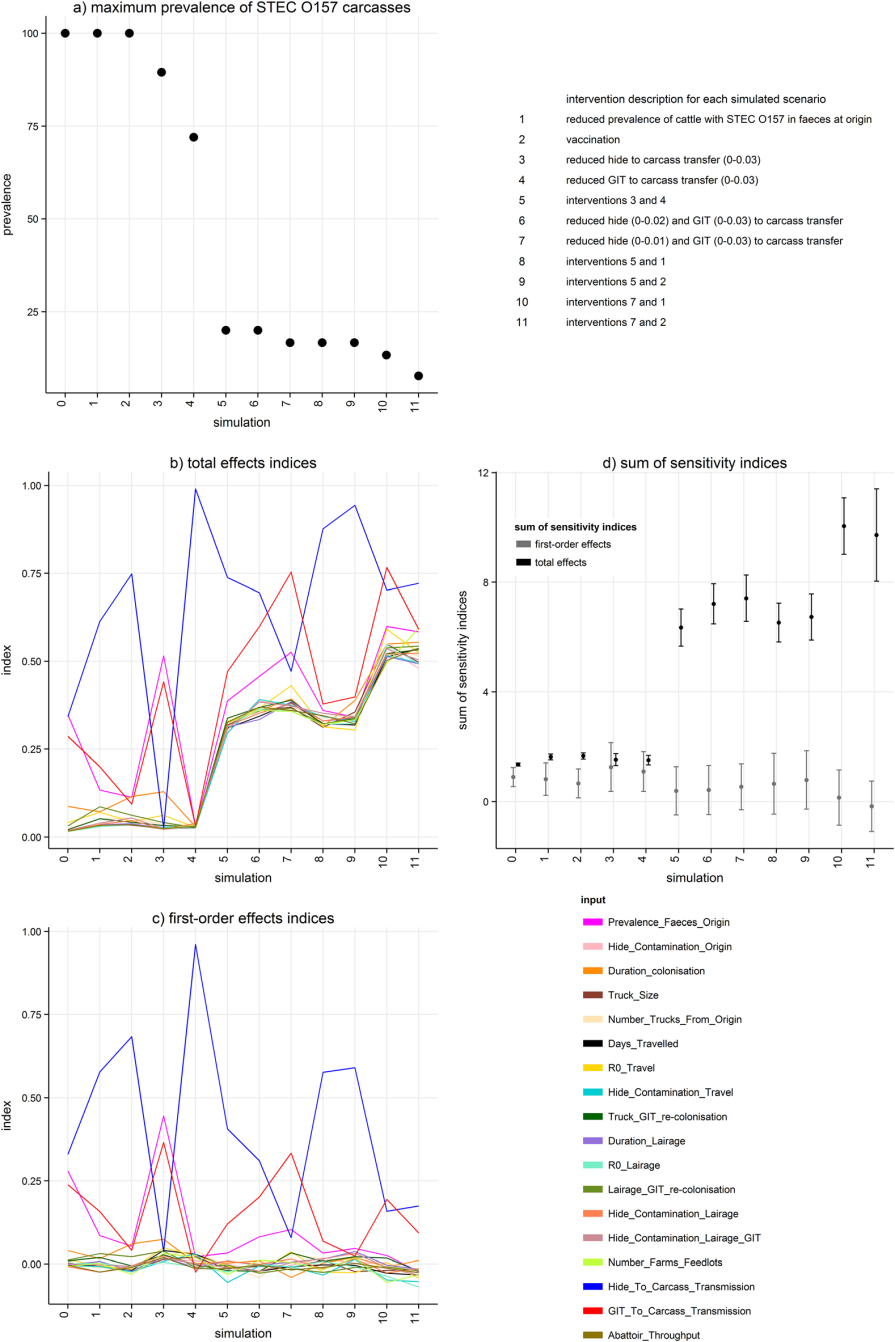
Maximum prevalence of STEC O157 contaminated carcasses (a), total effect (b) and first-order effect (c) sensitivity indices for all inputs, and sum sensitivity indices (d) during simulations of a model to determine the most effective intervention strategies to reduce the prevalence of *Escherichia coli* O157 (STEC O157) contamination of beef carcasses. In plot (d), bars indicate 95% confidence intervals.

The lower limit of the range of prevalence of STEC O157 contaminated carcasses was zero for all simulations in this study; therefore, we report the upper limit for each simulation only (maximum possible prevalence of STEC O157 contaminated carcasses). The maximum possible prevalence of STEC O157 contaminated carcases, sums of sensitivity indices (SIs), and the total and first-order effect SIs for inputs for all simulations are shown in Fig 4. During the initial simulation in which parameters were set at maximum range (Table 1), maximum possible carcass contamination prevalence reached 100% (Simulation 0, Fig 4a). The inputs with the greatest influence on variance of prevalence in this simulation were hide and GIT transmission to carcass, prevalence of STEC O157 in faeces at origin, and to a lesser extent, the duration of colonisation and *R*_*0*_ during travel (Simulation 0, Fig 4b and 4c). Although hide to carcass transmission had the greatest first-order effect (influence on carcass prevalence variance without other inputs), the sum of total effect SIs of inputs in the initial simulation (1.35, 95% confidence interval (CI) 1.30–1.41) indicated that interactions between inputs influenced output variance in this simulation; Fig 4c shows that the total effects of both prevalence of STEC O157 in faeces and the probability of hide to carcass transfer were equally important.

Simulations 1–4 introduced single interventions by reducing parameter ranges associated with the inputs with the highest SIs during the initial simulation. Simulations with reduced prevalence of STEC O157 in faeces at origin (Simulation 1) and vaccination (Simulation 2) did not reduce maximum possible prevalence of STEC O157 contaminated carcasses. Additional test simulations (results not shown) demonstrated that further reduced parameter ranges for prevalence of STEC O157 in faeces (0–10% and 0–1%) resulted in reduced maximum prevalence of contaminated carcases (87.7% and 83.2% respectively), indicating a non-linear relationship between this input and the prevalence of contaminated carcasses. Total and first-order effect SIs for Simulations 1 and 2 were similar; the influence of transfer of STEC O157 from hide to carcass on output variance became relatively more important than other inputs. During Simulation 3, reduction of the range of probability of transfer from hide to carcass reduced maximum carcass STEC O157 prevalence to 89.5%. The relative importance of hide to carcass transfer on carcass prevalence variance was reduced, whilst the importance of both GIT to carcass transfer and prevalence of STEC O157 in faeces at origin were increased. In Simulation 4, in which the probability of transfer of STEC O157 from GIT to carcass was targeted by intervention, the maximum carcass prevalence was reduced to 72%. During Simulation 4 the probability of transfer of STEC O157 from hide to carcass was responsible for nearly all output variance.

Combinations of interventions were introduced in subsequent simulations (5–11) based on results from the previous simulations. When the probability ranges of transfer from both GIT and hide to carcass were reduced concurrently (each 0–0.03; Simulation 6), maximum possible carcass prevalence was reduced to 20%, greater than the reduction in range expected due to an additive effect alone. Hide to carcass transfer remained as the input with the highest total and first-order effect SIs, followed by GIT to carcass transfer then prevalence of STEC O157 in faeces at origin. Although other inputs had relatively small first-order effects (Fig 4 (c)), their effect on the variance of carcass contamination prevalence in combination with other outputs (total effect SIs, Fig 4 (b)) increased so that the sum of total effects was 6.35 (95% CI 5.67–7.02, Fig 4 (d)), indicating that interactions had relatively more influence on the variance of carcass contamination prevalence, once hide and GIT to carcass transfer ranges were both reduced. During Simulations 6 and 7 the range of probability of hide to carcass transfer was reduced further (0–0.02 and 0–0.01 respectively). GIT to carcass transfer and prevalence of STEC O157 in faeces become more influential once the maximum probability of hide to carcass transfer was 0.01, and during this latter simulation, the maximum carcass contamination prevalence was reduced to 16.6%.

Introduction of either reduced prevalence of STEC O157 in faeces (Simulation 8) or vaccination (Simulation 9) with transfer of both hide and GIT to carcass at a range of 0–0.03 (instead of reduction of hide to carcass transfer range to 0–0.01) did not achieve lower output than Simulation 7. In these simulations, transfer from hide to carcass remained the most influential input on the maximum carcass contamination prevalence.

The final simulations introduced interventions to reduce prevalence of STEC O157 in faeces at origin (Simulation 10) or vaccination (Simulation 11) whilst the probabilities of hide and GIT to carcass transfer were 0–0.01 and 0–0.03, respectively. During Simulation 10, both GIT to carcass and hide to carcass transfer had the greatest influence on the prevalence of carcass contamination (maximum prevalence 13.6%). The relative importance of all other inputs during this simulation was increased and the sum of total effect indices was 10.0 (95% CI 9.02–11.08). During Simulation 11, hide to carcass transfer had the greatest influence on the prevalence (maximum prevalence 7.7%). The relative importance of all other inputs during this simulation was also high, and the sum of total effect indices was 9.7 (95% CI 8.03–11.41). Fig 4 (d) shows that confidence intervals of the sum of total effect SIs were sufficiently narrow to demonstrate increased interactions throughout the simulations.

## Discussion

In this study, combinations of interventions based on reducing the transfer of STEC O157 from hide and GIT to carcass were the most effective method of decreasing the maximum prevalence of STEC O157 contaminated carcasses. There were two key findings that contributed to this outcome. Firstly, global sensitivity analysis (GSA) using the Saltelli method identified that the probability of transfer of STEC O157 from both the gastro-intestinal tract (GIT) and hide to carcasses in the abattoir were the most influential inputs in this model, followed by the prevalence of cattle with STEC O157 in faeces at origin. This suggests that these are important drivers of the prevalence of STEC O157 contaminated carcasses. This finding was not unexpected—contamination of carcasses from hide or GIT in the abattoir are final transfer points for STEC O157 in the chain of events to carcass production, and elimination of transfer here prevents carcass contamination by this route. The prevalence of cattle with STEC O157 in faeces was also expected to be an important driver of carcass contamination. As well as reducing hide contamination at origin, opportunities for GIT colonisation and hide contamination beyond the farm gate are dependent on environmental sources of STEC O157 in the absence of already infected cattle. Whilst not preventing the contamination of carcasses, elimination of GIT colonisation on farm is likely to markedly reduce the prevalence of contaminated carcasses. However, elimination of either of these events (colonisation and transfer in the abattoir) is unlikely to be feasible; therefore, of particular interest were the parameter values required to achieve reduction in the prevalence of STEC O157 contaminated carcasses in this study and whether they are achievable in the field.

Due to a non-linear relationship between the prevalence of STEC O157 in faeces and on carcasses, interventions in this study that reduced maximum prevalence of STEC O157 in faeces to 30% were insufficient to reduce maximum possible prevalence of carcass contamination. Field efficacy of these interventions—for example, probiotics in feed and vaccination—is uncertain [2, 50]. Our upper limit of 30% prevalence was based on applying interventions to a herd with a potentially high initial prevalence of cattle with STEC O157 in faeces, such as that described by Williams et al. [35], and could be considered conservative—the prevalence might be reduced further in herds in which the initial prevalence is lower. However, although the median prevalence of contaminated carcasses produced by an abattoir is likely to be reduced if the prevalence of cattle with STEC O157 in faeces is less than 30% in some herds, this study demonstrates that the maximum possible prevalence of contaminated carcasses can still be high unless all herds that supply an abattoir attain low prevalence of STEC O157 in faeces. We consider this unlikely in abattoirs that receive cattle—particularly young stock—from multiple sources. Mixing of cattle at saleyards, travel rest stops, or during transport and lairage (steps not included in this model) would further limit the efficacy of partial uptake of these interventions by farms and feedlots. Therefore, targeting reduction in the prevalence of cattle with STEC O157 in faeces at origin as a single intervention is unlikely to consistently achieve a low prevalence of STEC O157 contaminated carcasses.

Single interventions that reduced the probability of transfer of STEC O157 from either hide or GIT to carcass within a range of 0–0.03 decreased the maximum possible prevalence of contaminated carcasses. Although information from previous studies suggest that these parameter values might be achievable in some abattoirs [46, 48, 56], it has also been suggested that the concentration of STEC O157 on hide or in faeces influences the probability of transfer; if an animal has a sufficiently high concentration of STEC O157 in faeces or on hide, hygiene practices in the abattoir could be overwhelmed, resulting in a higher probability of carcass contamination. For example, some cattle (sometimes termed “super-shedders”) excrete orders of magnitude greater numbers of STEC O157 in faeces than other cattle. Identification of high-shedding cattle has been the focus of previous research because of the suggestion that it is more difficult to prevent contamination of carcasses from these cattle [49]. However, recent longitudinal surveys in Australia found that high-shedding of STEC O157 in faeces was not predictably associated with specific individuals within cohorts of dairy and beef cattle [33, 35]. Also, rapid identification of high-shedding cattle or cattle with highly contaminated hides prior to slaughter is not currently feasible. Although a low probability of transfer appears to be achievable in some abattoirs, it might be more difficult to consistently achieve in abattoirs that receive a high proportion of high-shedding cattle, young cattle (in which a higher concentration of faecal STEC O157 is more likely in those that are shedding), or cattle with highly contaminated hides. We further address this point later in the discussion.

The second key finding from this study was that combinations of interventions achieved a much greater reduction in the prevalence of STEC O157 contaminated carcasses than that due to an additive effect of interventions. Saltelli GSA demonstrated that this was most likely due to interactions between inputs in the model (measured by the sum of total effect sensitivity indices). As the maximum possible prevalence of contaminated carcasses decreased due to improved combinations of interventions, the relative importance of interactions between all inputs increased. Therefore, the most effective combination of interventions was reduction of the probability of transfer from both hide and GIT to carcass and vaccination, because vaccination targets several inputs in the model (transmission between cattle and the probability of re-colonisation in the lairage and during travel, as well as the prevalence of STEC O157 in faeces at origin). The probability of hide to carcass transfer in this simulation remained the most influential input, despite a low range of 0–0.01. This is consistent with empirical studies which have suggested that the hide is the major source of carcass contamination at slaughter, compared to the GIT [48]. This suggests that in abattoirs that are already achieving a low prevalence of STEC O157 contaminated carcasses, additional reduction of prevalence can either be achieved through further decreasing the probability of transfer from hide, or through implementation of a combination of interventions that target a range of pre-abattoir factors such as contamination and colonisation from environmental sources as well as other cattle prior to the abattoir. Although the efficacy of combinations of interventions has been identified previously [17, 19], this study provides additional information by demonstrating that the efficacy of combinations of interventions in the beef-harvest chain relies on reduction of transfer of STEC O157 in abattoirs.

There are many sources of uncertainty in simulation models that can limit findings. In the initial simulation we used wide parameter ranges to account for uncertainty associated with some inputs in this model. Inputs other than the probabilities of transfer of STEC O157 from hide and GIT to carcasses and the prevalence of cattle with STEC O157 in faeces only became important once the prevalence of contaminated carcasses was very low. Further reduction of the prevalence of contaminated carcasses provided by interventions that target these inputs would be relatively small; therefore, this study suggests that further research to reduce uncertainty about these inputs is of low priority.

In addition to uncertainty associated with input parameters, model output is dependent on structure and accuracy of processes between stages. This study identified that the most important intervention point was transfer from hide and GIT to carcasses in the abattoir, but did not examine the effect of concentration of STEC O157 on hides or in faeces on the probability of transfer. Concentrations of STEC O157 on hides or in faeces were not included due to insufficient comparable information (studies were from heterogeneous populations or used different laboratory methods) to accurately model either input parameters or processes affecting STEC O157 concentrations between stages in the beef-harvest chain. Interventions to reduce STEC O157 transfer from hide and GIT to carcasses include “spear-cutting" (the hide is incised from the inside towards the external surface), rotating knives and immersion of used knives in hot water, downward hide-pullers and dehairing or spray-washing of carcasses prior to de-hiding [19, 46, 57, 58]. The relative effects of these interventions in practice on both the prevalence and distribution of STEC O157 on carcasses compared to the prevalence and distribution of STEC O157 on hides and in faeces is uncertain. It is possible that these interventions only decrease the prevalence of carcasses with a low level of contamination and that the prevalence of carcasses with high levels of contamination (such as those that could be associated with high-shedders) remains the same; this outcome might have little impact on reducing the incidence of STEC O157 associated illness in humans. Therefore, this is an important information gap and further empirical research in the field is required to investigate the relationship between the prevalence and distribution of STEC O157 concentration on hides, in faeces and on carcasses in the slaughter-line and how they are affected by interventions. Interventions that reduce faecal and hide concentration of STEC O157 –for example, vaccination and washing carcasses prior to de-hiding—could be essential to achieve low probability of transfer from hide and GIT to carcasses in abattoirs.

Both direct and indirect (from environmental sources) transfer of STEC O157 between cattle were modelled in this study. Whilst most ranges for parameters used in the initial maximum-range scenario in this study were wide, the ranges for *R*_0_ –used to simulate direct transmission of STEC O157 between cattle during transport and lairage—were particularly generous. We used a high upper range for *R*_0_ to reflect high prevalence of STEC O157 in faeces observed in recent empirical studies and the possibility—even if remote—that this could be due to a high level of direct transmission. Although the relative importance of each method of transfer is difficult to assess empirically, recent modelling studies have suggested that transfer from the environment is an important driver of prevalence of STEC O157 in faeces [43, 44]. In this study, prevalence of STEC O157 contaminated carcasses was not sensitive to the high range of *R*_0_. Whilst this neither supports nor contradicts the importance of direct transmission on the prevalence of STEC O157 in faeces or its importance to on-farm control, it suggests that determining the relative importance of direct and indirect transmission is not an information gap required to achieve effective interventions to reduce the prevalence of contaminated carcasses.

The Saltelli method used for sensitivity analysis in this model gave a number of advantages over methods used previously for sensitivity analysis of STEC O157 contamination of carcasses or STEC O157 associated illness in humans. Saltelli GSA is robust to both non-linear and non-montonic relationships between model inputs and output [59]. In this study, a non-linear relationship was apparent between the prevalence of STEC O157 in faeces (at origin) and on carcasses, and it is possible that other non-linear relationships exist in this model that were not identified. Sensitivity analysis using methods that assume model linearity—for example correlation coefficients—could have resulted in biased estimates of the importance of inputs on the prevalence of contaminated carcasses. Saltelli GSA can also identify and quantify interactions between inputs. Interactions were extensive once the probability of transfer of STEC O157 transfer in the abattoir was reduced. This is an important insight that would not have been detected with other commonly used methods for sensitivity analysis such as “one at a time” analysis [23]. Finally, this study demonstrated how Saltelli GSA can be used iteratively to systematically direct introduction of intervention strategies into models to achieve effective intervention strategies. This provided a more comprehensive analysis of potential interventions than either sensitivity analysis of a single simulation or *ad hoc* introduction of interventions. Limitations of variance-based GSA include the computational power and time required to run sufficient iterations to provide estimates with narrow confidence intervals. Although iterations in this study were sufficient to estimate first-order and total effect indices, it is possible to estimate the influence of pairwise interactions between inputs (second-order effects). Although this could provide further insight about the most effective combination of pre-abattoir interventions in the final simulation in this model, the number of iterations required and computational limitations made this infeasible in this study.

## Conclusion

This study used a computational model and the Saltelli method for global sensitivity analysis and provided insight about the importance of interactions between inputs in the beef harvest chain as well as identifying the factors with the greatest influence on the prevalence of STEC O157 contaminated carcasses. This is a valuable practical addition to the findings of existing studies, indicating that effective reduction of the prevalence of STEC O157 contaminated carcasses relies on reduction of the probability of transfer of STEC O157 from hide and GIT to carcasses in the abattoir. Other interventions such as vaccination can provide further, smaller reductions in the maximum prevalence of contaminated carcasses. The most important information gap identified in this study was the effect of distribution of concentrations of STEC O157 on hides and in faeces on the probability of transfer of STEC O157 to carcasses, and the resulting distribution of concentrations of STEC O157 on carcasses. Understanding these points is essential to assess the effect of interventions at this stage in the harvest chain and evaluate their potential benefit in reducing the risk of STEC O157 associated illness in humans.
